# Annotations

**Published:** 1908-07-25

**Authors:** 


### ANNOTATIONS.

### The Cause of Sunstroke.

Of all the devastating and mysterious calamities to which flesh is heir, there are few which have so successfully defied explanation as sunstroke. Pre-eminently a malady of tropical climates and of Europeans, it occurs also in the most temperate zones. As with other diseases, there are those who seem to be practically insusceptible, and can court without danger exposure which would certainly mean immediate siriasis to the majority of their fellows; and there are also those from whom the most elaborate precautions fail always to ward off an attack. There are at least four hypotheses of the causation of sunstroke, as Lieut.-Col. A. Duncan has lately pointed out. The most interesting of these, but the least probable as far as can at present be seen, is the microbic theory of Dr. Sambon; the possession of immunity is easily explained by it, but there are objections which are as yet insuperable. Dr. Duncan prefers to the toxic and the caloric that known as the actinic hypothesis, which assigns to the actinic rays and to them alone the power of affecting the nervous system so as to cause sunstroke. He prefers, therefore, a red or orange lining for the helmet or other head-covering to the green colour commonly supplied by those who equip our equatorial exiles, and advocates a spine pad of the same colour. The general trend of opinion among those who are qualified by personal experience to judge this matter seems decidedly, though not unanimously, in favour of this plan, though there are still many who draw a distinction between sunstroke and heatstroke, and would regard them as two separate diseases. Certain it is that even in this country there are, during a hot summer, cases of sudden illness to which no origin other than a climatic one can be assigned, and which do not conform strictly to any variety of undoubted sunstroke as seen in India. The difficulties of research into this disease are considerable, but we have learnt to regard no problem of tropical medicine as insoluble, and it is much to be hoped that our knowledge of sunstroke may soon be greatly extended.

### The Communion Cup.

A recent letter by Dr. David J. Morgan, medical officer of health for the Borough of Swansea, addressed to the governing bodies of the various places of Divine worship in Swansea, draws attention once more to a singular anomaly of civilised life at the present day. The matter has often been dealt with by medical journals, but the education of public opinion is slow and laborious. Dr. Morgan refers to the fact that in the celebration of the Holy Communion 50 or 100 persons often drink out of one and the same cup. In this way the spread of diseases of the nose, mouth, throat, and lungs is favoured. He urges the necessity of providing separate drinking vessels for each communicant, as is now done in many parts of the country. The vessels, according to Dr. Morgan, are made either of glass or metal, and can be thoroughly cleansed with boiling water after use. They are made in small sizes, arranged in circular manner on a tray with raised platforms. There is no reason, he says, why we should sanction in public places methods which would not be tolerated for a single moment in our own houses, and indeed he is perfectly right. Nevertheless, old-established customs die very hard, and when they are bound up with feelings of genuine reverence their downfall at the hands of hygiene is a difficult process.

### Government Research.

An interesting list has been issued of eight subjects of research for which the President of the Local Government Board has authorised grants from the fund annually voted by Parliament in aid of the scientific investigation of disease. Dr. M. H. Gordon is to inquire further into the micro-organisms found in the throats of scarlet fever patients, presumably in the hope of finding the pathogenic organism of that disease, and the best way of treating the septic complications. Drs. Theodore Thomson and C. J. Thomas investigate protracted and recurrent infection in diphtheria, another subject with highly practical bearings. A similar research on enteric fever is to be undertaken by Drs. Thomson and Hedingham; on this, as is well known, a good deal of evidence, which needs correlation and extension, has already accumulated. Two problems connected with the purity of milk will be attacked by Dr. W. G. Savage and Professor Delépine respectively: the former is occupied by the bacteriological measurement of pollution of milk and by the study of paratyphoid bacilli in man and of streptococci in goats, and the latter will codify the results of the bacteriological examination of over seven thousand samples of milk from various parts of the country. Dr. Copeman and Professor Nuttall will investigate the part played by flies as carriers of disease, but it is not stated whether the scope of their research includes sunnier countries where fly plagues are many times more prevalent than here. Another subject the threshing out of which should prove instructive is allotted to Dr. Farrar; that is the bacteriology and biology of bedding, the somewhat alliterative title of a most important matter. The relation of flock beddings to vermin will form a considerable portion of this inquiry. Yet another subject of great interest, with which Dr. Basil Cook will be employed, is the social incidence of diseases, particularly the prevalence of varicose veins and hernia under different social conditions. It will be seen that the list comprises problems of the utmost diversity and of far-reaching importance. It is announced that it is not yet complete, for further subjects are to be made public at a later date: the amount of success which will attend the various researches remains, of course, to be seen, but from the extremely practical selection that has so far been made we may draw a good augury of practical results.

